# Effects of Continuous Positive Airway Pressure Treatment on Vascular Function in a Real-Life Cohort of Elderly Patients with Obstructive Sleep Apnoea Syndrome

**DOI:** 10.3390/biomedicines12112563

**Published:** 2024-11-08

**Authors:** Mara Volpentesta, Valentino Condoleo, Alberto Panza, Giandomenico Severini, Luca Soraci, Cataldo Rotondo, Giuseppe Armentaro, Corrado Pelaia, Vanessa Teresa Fiorentino, Francesco Andreozzi, Giorgio Sesti, Andrea Corsonello, Angela Sciacqua

**Affiliations:** 1 Unit of Geriatric Medicine, IRCCS INRCA, 87100 Cosenza, Italy; mara.volpentesta@yahoo.it (M.V.); l.soraci@inrca.it (L.S.); a.corsonello@inrca.it (A.C.); 2 Geriatrics Division, University Hospital “R. Dulbecco”, 88100 Catanzaro, Italy; condoleovalentino@gmail.com (V.C.); giuseppearmentaro91@gmail.com (G.A.); sciacqua@unicz.it (A.S.); 3 Department of Medical and Surgical Sciences, University Magna Graecia of Catanzaro, 88100 Catanzaro, Italy; albertopanza94@libero.it (A.P.); pelaia.corrado@gmail.com (C.P.); vanessa.fiorentino@unicz.it (V.T.F.); andreozzif@unicz.it (F.A.); 4 Coordinamento Medico legale, Istituto Nazionale della Previdenza Sociale, 88100 Catanzaro, Italy; crotondo63@gmail.com; 5 Department of Clinical and Molecular Medicine, University of Rome-Sapienza, 00185 Rome, Italy; giorgio.sesti@uniroma1.it; 6 Department of Pharmacy, Health and Nutritional Sciences, University of Calabria, 87100 Rende, Italy

**Keywords:** CPAP, elderly, OSAS, arterial stiffness, endothelial dysfunction

## Abstract

Background: Obstructive sleep apnoea syndrome (OSAS) is an independent risk factor for cardiovascular morbidity and mortality and has a detrimental effect on vascular function, in particular on arterial stiffness and endothelial function. Continuous positive airway pressure (CPAP) is the gold-standard therapy for OSAS and its effects on arterial stiffness and endothelial function have been demonstrated in non-elderly patients. Objectives: The objective of this study was to evaluate the effect of one year of CPAP treatment on arterial stiffness, through assessment of carotid–femoral pulse wave velocity (cf-PWV), and on endothelial function, through the reactive hyperaemia index (RHi), in a real-life cohort of elderly patients with moderate-to-severe OSAS and several comorbidities. Methods: In this nonrandomised prospective study, we enrolled 469 consecutive elderly patients affected by moderate-to-severe OSAS distributed in two groups: CPAP-treated (*n* = 225) and untreated patients (*n* = 244). Results: At one-year follow-up, in the treated group emerged an important improvement in poligraphics (AHI, ODI, TC90, mean SpO_2_%), laboratory (HOMA index, eGFR, hs-CRP) and vascular function parameters: cf-PWV. The stepwise multivariate linear regression demonstrated a significant correlation between the delta of the polygraph parameters and the delta of PWV and RHi. Conclusions: Our study confirmed the favourable effects of CPAP therapy in a cohort of elderly patients affected by OSAS and several comorbidities on sleep respiratory parameters and vascular function; early diagnosis and treatment with CPAP might be beneficial to delay or prevent the occurrence of cardiovascular events in these groups of patients.

## 1. Introduction

Obstructive sleep apnoea syndrome (OSAS) consists of repetitive episodes of upper airway (UA) collapse that cause complete (apnoea) or partial (hypopnoea) intermittent airflow obstruction during sleep. The estimated prevalence of OSAS is approximately 5–15% in the general population and affecting up to 33.3% of elderly people in the eighth decade of life and 39.5% in the ninth decade; but risk factors, symptoms and natural history differ from those in middle-aged patients [1,2,3,4]. OSAS is considered an independent risk factor for cardiovascular disease. UA collapse leads to chronic intermittent hypoxemia (CIH), hypercapnia, intrathoracic pressure fluctuations and sleep fragmentation that, in turn, increase oxidative stress and systemic inflammation and cause sympathetic and renin–angiotensin–aldosterone system (RAAS) hyperactivity [5]. These pathogenic mechanisms are believed to induce arterial stiffness augmentation and endothelial dysfunction, which are early vascular damage markers associated with increased cardiovascular risk [6,7]. Further, it is widely accepted that age is a major determinant of increased arterial stiffness and endothelial dysfunction [8,9]. Prior research has indicated a correlation between obstructive sleep apnoea syndrome (OSAS) and a reduction in arterial stiffness, irrespective of alterations in blood pressure. Furthermore, in a cohort of elderly patients with sleep breathing disorders, a notable correlation was observed between blood pressure/pulse rate and endothelial function parameters. In younger patients, CPAP therapy demonstrated a favourable impact on endothelial function [10]. In light of these observations, the aim of this study was to evaluate the effects of one year of CPAP treatment on arterial stiffness, as measured by carotid–femoral pulse wave velocity (cf-PWV), and on endothelial function, as determined by the reactive hyperaemia index (RHi), in a cohort of elderly patients affected by moderate-to-severe OSAS and several comorbidities, not included in previous studies.

## 2. Materials and Methods

### 2.1. Study Population

The present study, which was prospective, observational and not randomised, enrolled 469 consecutive patients aged ≥ 65 years affected by moderate or severe OSAS who had several comorbidities and were referring to the Geriatric Division of the University “Magna Graecia” in Catanzaro from October 2012 to September 2023, in the Catanzaro Metabolic Risk Factors (CATAMERI) Study. Inclusion criteria were the following: patients aged ≥ 65 years, able to give written consent and affected by moderate or severe OSAS with or without respiratory failure, with indications to nocturnal CPAP, eventually enriched with oxygen therapy. Exclusion criteria were the presence of central or mixed apnoea syndrome, severe dementia or Alzheimer’s disease, prior ischemic or haemorrhagic major stroke, psychiatric comorbidities or drug therapies that may affect respiratory function or sleep and common contraindications to nocturnal CPAP, recent facial or upper airways/aesophagogastric surgery, anatomical alterations or facial trauma and difficulty in swallowing, detected by medical history. At the time of enrolment and after one year, patients underwent clinical interview, physical examination, sero-haematological exams, overnight polygraphy and instrumental determination of vascular function. The Epworth sleepiness scale (ESS) questionnaire was administered to all participants at diagnosis and after one year. At baseline, patients underwent an ear, nose and throat (ENT) visit to evaluate Mallampati score and exclude surgical indications. After preliminary evaluations, patients started nocturnal CPAP titration using a ResMed Airsense 10 Autoset (Resmed, Sydney, Australia) in auto-CPAP mode with default minimum–maximum pressure settings at 4–20 cm H_2_O. Humidification and choice of interface were made on an individualised patient basis. Patients who well adapted to therapy and reached a mean use time ≥ 4 h/night were assigned to the group of CPAP-treated patients (*n* = 225), while patients who interrupted titration early, did not adapt well or chose to receive best support care were included in the untreated group (*n* = 244). BSC was provided through advice on sleep hygiene and weight loss, as appropriate to each patient. Medical appointments were scheduled for all patients at 1 year after diagnosis to ensure the same number of medical visits in all patients (with or without CPAP). Evaluation of adherence to CPAP therapy was objectively assessed by reading the device’s smart card from the start of treatment to the end of the follow-up. Patients were classified as being adherent if the average cumulative compliance was ≥4 h/day.

### 2.2. Laboratory Determinants

All laboratory measurements were carried out on peripheral blood samples after at least 12 h of fasting. Glycaemia was determined by the glucose oxidase method (glucose analyser, Beckman Coulter, Milan, Italy). Creatinine levels were measured using the Jaffé method. The estimation of glomerular filtration rate (eGFR) was based on the new CKD-EPI (Chronic Kidney Disease Epidemiology Collaboration) equation [11]. Serum uric acid (UA) levels were assessed using the URICASE/POD method (Boehringer Mannheim, Mannheim, Germany). Serum levels of high-sensitivity C-reactive protein (hs-CRP) were measured by an immune-turbidimetric method automated system (Cardio Phase hs-CRP, Milan, Italy). In addition, glycated haemoglobin (HbA1c) was measured by high-performance liquid chromatography certified by the national glycohaemoglobin standardisation program (NGSP) and using an automatic analyser (Adams HA-8160 HbA1c analyser, Menarini, Italy). Analytical determinations were taken using an automatic particle counter (Siemens Healthcare Diagnostics, ADVIA 120/2120 Haematology System, Milan, Italy) to measure haemoglobin, haematocrit and white blood cell count. Plasma concentrations of insulin were determined by chemi-luminescence test (Roche Diagnostics, Mannheim, Germany). Insulin resistance was determined by the homeostasis model assessment (HOMA) index [12].

### 2.3. OSAS Diagnosis

OSAS diagnosis was made after one-night polygraphy that included recordings of nasal flow, chest and abdomen movements and oxygen saturation. The report was analysed with dedicated software by two expert operators blinded to patients’ characteristics, according to 2017 American Academy of Sleep Medicine (AASM) guidelines [13]. Referring to the number of apnoea plus hypopnea events per hour of sleep (AHI, e/h), OSAS was defined as moderate with AHI between 15 and 29.9/h and severe when ≥30/h. We also evaluated the average nocturnal saturation (mean SpO_2_, %), the oxygen desaturation index (ODI, e/h) corresponding to the number of episodes of desaturation > 3% per hour of sleep and the percentage time of saturation <90% (TC90, %). A value of TC90 > 30% allows the diagnosis of nocturnal respiratory failure (NRF).

### 2.4. Vascular Function Parameters Measurement

Arterial stiffness measurements were obtained by a validated system (SphygmocorTM; AtCor Medical, Sydney, Australia) that employs high-fidelity applanation tonometry (Millar) and appropriate computer software for the analysis of pressure waves (SphygmocorTM), as previously reported. Aortic PWV was determined by setting the distance between the surface markings of the mandibular angle, the sternal notch and the femoral artery as the path length between the carotid and femoral arteries (L) and measuring the time of propagation (DT) of the pressure waveform through this segment (L/DT) [14]. Endothelial function was assessed using EndoPAT (Itamar Medical, Caesarea, Israel), a noninvasive device based on the plethysmography method that uses the peripheral response to reactive hyperaemia to evaluate endothelial function. The device records endothelium-mediated changes in the digital pulse waveform known as the PAT (peripheral arterial tone) signal, measured through a special finger probe on the index finger of the two hands; after that, hyperaemia was induced by occluding blood flow through the brachial artery for 5 min using an inflatable cuff on one hand. The amplitude of the induced hyperaemia is used to calculate RHi [15].

### 2.5. Statistical Analysis

Continuous data were expressed as mean ± standard deviation (SD) or as the median and interquartile range when appropriate. Normally distributed data were analysed using *t*-test for paired data, while not normally distributed data were analysed by the Mann–Whitney test. Subsequently, in the group of CPAP-treated patients, a simple linear regression model was built with the delta (Δ, i.e., changes) between follow-up and baseline in PWV and in RHi as the dependent variable and Δ of the other clinical variables considered to be major as independent variables. Therefore, changes in the variables that significantly correlated with changes in the dependent variable were entered into a stepwise multivariate linear regression model. Statistical analysis was carried out using the SPSS V20.0 program for Windows (SPSS Inc., Chicago, IL, USA).

## 3. Results

**Figure 1:**
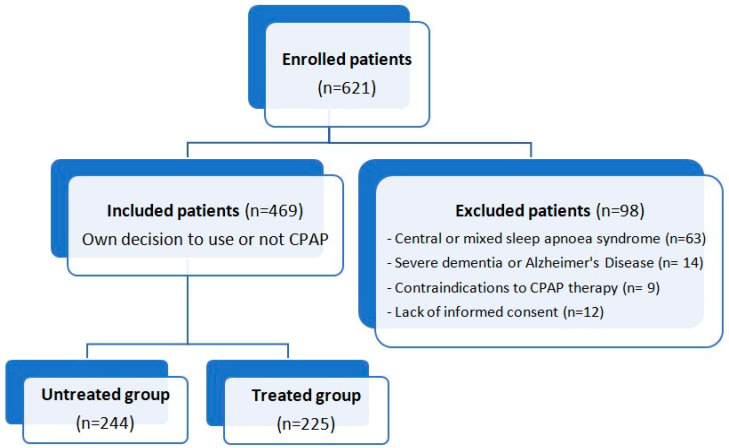
Flowchart of the recruitment process of the study.

From an initial cohort of 621 patients, 12 were excluded because they did not sign the informed consent, 14 because they were affected by dementia, 9 because they had contraindications to CPAP, and 63 because they had central or mixed apnoea syndrome (Figure 1).

The final cohort included 469 patients, 70.4% men and 29.6% women, with a median age of 74.8 ± 5 years. In particular, 47.9% were affected by moderate OSAS while 52% by severe OSAS and 28.1% were complicated by NRF. In the population, 77.8% were affected by arterial hypertension, 55.2% of patients suffered from type 2 diabetes mellitus, 44.1% from chronic kidney disease (eGFR <60 mL/min/1.73 m^2^), 12.6% from chronic heart failure (CHF), 22.6% from ischemic heart disease (IHD) and 36% from chronic obstructive pulmonary disease (COPD) or asthma. At baseline, statistically significant differences only emerged between the two studied groups for smoking habit and dyslipidaemia (Table 1).

After seven days of CPAP titration, only 47.9% adapted to CPAP and chose to continue therapy and best support care (BSC) (*n* = 225); the remaining patients entered the untreated group and received BSC (*n* = 244). Finally, treated patients presented significantly higher Mallampati scores (2.9 ± 0.9 vs. 2.4 ± 1.0 in untreated group, *p* = 0.0001). Only 20% of treated patients needed oxygen supplementation. In the untreated group, the mean AHI was 36.7 ± 15.5/h versus 37.5 ± 17.7/h in the treated group, *p* = 0.576 (Table 2).

**Figure 2:**
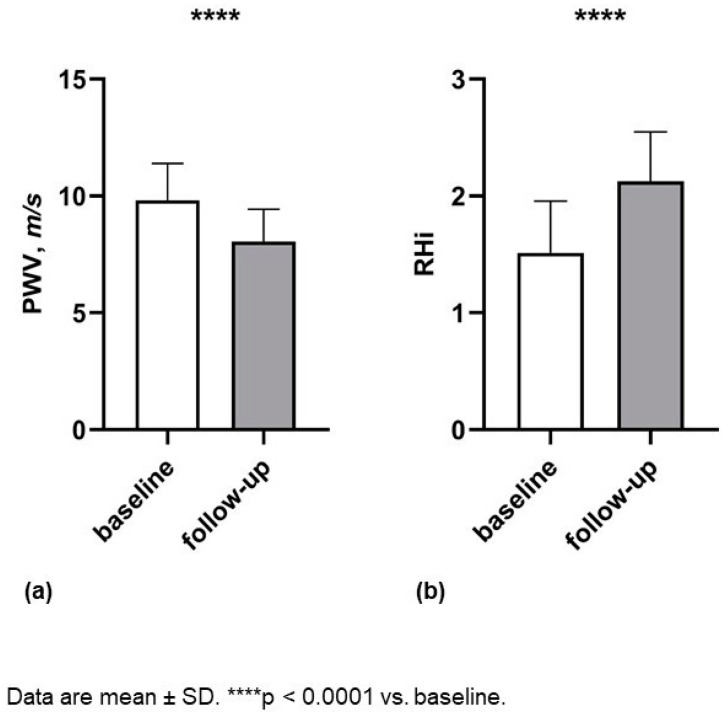
Changes in vascular parameters, (**a**) PWV, (**b**) RHi in treated group between baseline and follow-up.

In the CPAP group, after one-year follow-up, there was an important improvement in respiratory, some laboratory and vascular function parameters. No differences emerged in BMI, probably because the untreated group strictly followed life-style advice established in BSC. No statistically significant differences emerged between the two groups among vascular function parameters at baseline (Table 3); after one year of treatment with CPAP, a significant improvement was obtained in all considered parameters, in particular, in PWV (−1.0 (−1.7/−0.8) vs. −1.6 (−2.1/−1.3) m/s, *p* < 0.0001) and RHi (0.4 (0.3/0.5) vs. 0.6 (0.5/0.7), *p* < 0.0001) (Table 4) (Figure 2).

A comparison of the changes in polygraphic parameters between the two groups revealed a significant difference between the CPAP therapy and the control groups (Table 5). In the treated group, linear regression analysis revealed a statistically significant relationship between ΔPWV and ΔRHi and the major considered clinical variables (Table 6).

By using Δ PWV as a dependent variable, PWV changes were significantly associated with variations in AHI, ODI, eGFR, uric acid and hs-CRP. Variations in PWV correlated significantly and directly with changes in poligraphic parameters, uric acid and hs-CPR and inversely with eGFR changes. The same parameters, except for eGFR and with contributions of ESS, TC 90 and haematocrit (HCT) changes, were significantly associated with Δ RHi. Variations in RHi correlated significantly and inversely with poligraphic parameters, ESS score, uric acid, hs-CRP and HCT changes. Furthermore, variables that showed a significant correlation with changes in Λ PWV and Λ RHi were included in a stepwise multivariate linear regression model to identify the independent predictors of these variations. We observed that changes in AHI, ODI, hs-CRP, uric acid and eGFR contributed, respectively, to 35.8%, 10.4%, 5.5%, 2.5% and 1.2% of the variability in PWV, with the whole model accounting for 55.4% of PWV variations (Table 7). On the other side, considering RHi variations, we observed that changes in uric acid, AHI, ODI, hs-CRP, ESS score, HCT and TC90 contributed, respectively, to 27.0%, 13.0%, 7.0%, 2.0%, 1.0%, 0.7%, and 0.2% of the variability, with the whole model accounting for 50.9% of RHi variations.

## 4. Discussion

In a real-life cohort of elderly patients affected by moderate-to-severe OSAS and other important comorbidities, we observed that CPAP treatment with a compliance ≥ 4 h/night significantly improved vascular function parameters, in particular PWV and RHi, after one year of treatment. According to previous studies, we confirmed the positive effect of CPAP on poligraphic parameters (AHI, ODI, TC90, mean SpO_2_), renal function (eGFR), systemic inflammation (hs-CPR) and metabolic status (HOMA index and uric acid) [16,17,18]. In our population, baseline characteristics of PWV and RHi did not significantly differ between the two studied groups, but at follow-up, statistically significant differences emerged. These findings are a result of the known effects of CPAP, which corrects sleep-related respiratory events and associated CIH, reduces the mechanical stress on the vasculature caused by pressure fluctuations during apnoea and interferes with the mechanism involved in the pathogenesis of AS and endothelial dysfunction. A statistically significant association was found between improved PWV and reduced respiratory apnoea events (AHI and ODI), metabolic parameters (uric acid), improved renal function (eGFR) and inflammatory markers (hs-CRP) in the treated group. More specifically, simple and multivariate linear regression analysis showed that improvements in AHI and ODI largely explained the variation in PWV, and reductions in uric acid, AHI and ODI correlated with the changes in RHi after one year of treatment with CPAP. The reduction in AHI and ODI explained 46.2% of PWV changes and the reduction in uric acid, AHI and ODI explained 47% of RHi variations. It is widely accepted that uric acid, eGFR and hs-CPR are the main determinants of arterial stiffness and endothelial dysfunction [19,20,21]. The reported reduction in uric acid and hs-CPR due to the effect of CPAP on CIH contributed to the improvement of both PWV and RHi by further reduction in oxidative stress, inflammation and insulin resistance. Uric acid has been established as a major predictor of PWV in a cohort of hypertensive patients, supporting a synergistic action between uric acid and HOMA index in inducing subclinical vascular damage, and the role of insulin resistance as a mediator by which uric acid increases AS. The mechanisms by which this occurs have been identified through experimental studies. They involve interference with insulin signalling, IGF-1 levels, and the activation of inflammatory processes and systems such as RAAS and the sympathetic system. These mechanisms promote oxidative stress and endothelial dysfunction [22,23,24]. However, in contrast with this evidence, in our study, changes in HOMA index did not significantly correlate with changes in PWV. In a previous study we conducted on obese patients affected by moderate or severe OSAS with normal renal function, CPAP therapy significantly improved eGFR after a follow-up of 6 months, and the main determinant of eGFR variations were the reductions in AHI and TC90; so in our study, the improvement in renal function in the CPAP-treated group represented one of the determinants of PWV variations. This finding supports the hypothesis that CKD and OSAS share common pathogenic mechanisms, including oxidative stress, metabolic alterations and sympathetic activity, and that resolution of respiratory events and CIH improve endothelial glomerular function [25,26,27]. A pathogenic role in renal function decline is also played by arterial stiffness; mineral metabolism disturbances, vascular calcifications, formation of advanced glycation end-products (AGEs) and acute and chronic volume overload occurring in ESRD and CKD patients have been proposed as the main pathogenic mechanisms linking arterial stiffness to renal disease. Changes in ESS score, TC90 and HCT were involved in j variations in RHi. The underlying pathogenic mechanism of sleepiness in OSAS includes CIH and repetitive arousals that result in sleep fragmentation and changes in neurons and the brain circuit involving noradrenergic and dopaminergic neurotransmission in wake-promoting regions of the brain [27]. In particular, sleep fragmentation itself may adversely affect endothelial function and CPAP stops this mechanism, improving both sleepiness and endothelial function [28]. Several studies have also found a significant association between variations in endothelial function and TC90. For instance, a study conducted in elderly patients found that brachial artery baseline diameter and flow-mediated dilation correlated more with TC90 than AHI, which confirms the pathogenic role of hypoxemia/re-oxygenation cycles [13]. The effects of HCT variations are probably due to the higher level of oxygenation reached with CPAP. The novelty of our study is that we considered a numerous group of elderly patients affected by several comorbidities as in real life. Moreover, our study population at baseline complained of mild sleepiness (ESS score < 16), but contrary to what emerged from other studies on AS, the positive impact of treatment was already evident [29]. Patients in the CPAP-treated group had a good compliance to therapy, not always reached in other studies. Finally, the follow-up was longer than other studies reported, confirming the persistence of positive effects after one-year follow-up.

### Limitations

The principal limitation of this study is its single-centred and nonrandomised design, which did not eliminate the potential for bias in participant selection. Furthermore, the inclusion of a matched control group would have been beneficial. Another limitation is the use of one-night polysomnography to make a diagnosis, which did not include electroencephalogram (EEG) recordings and did not allow for the study of the contribution of arousal, respiratory effort-related arousal (RERA), and fragmentation of sleep architecture. Additionally, the role of the number of hours spent complying with therapy in ameliorating arterial stiffness and endothelial dysfunction was not evaluated. Finally, other mechanisms involved in increasing AS and causing endothelial dysfunction, which OSAS exacerbates and on which CPAP can have an effect, such as sympathetic hyperactivity, were not considered.

## 5. Conclusions

Finally, this study confirmed the impact of CPAP therapy in a cohort of elderly patients affected by OSAS and several comorbidities on the improvements of sleep polygraph parameters, renal function and metabolic-inflammatory status and vascular function. CPAP therapy corrected sleep respiratory events and CIH, and the induced pathogenic mechanisms (oxidative stress, systemic inflammation, sympathetic hyperactivation and arousal, activation of RAA system) seemed to have a positive effect on renal function, metabolic parameters and inflammation markers and ameliorate arterial stiffness and endothelial function in elderly patients with OSAS and several comorbidities. In this context, an early diagnosis and treatment with CPAP might also be beneficial to delay or prevent the occurrence of cardiovascular events in these group of patients.

## Figures and Tables

**Table 1:** Comparison of comorbidities and pharmacotherapy of the study population between treated and untreated groups.

	Whole Population (*n* = 469)	Untreated Group (*n* = 244)	Treated Group (*n* = 225)	*p* *
Smokers, *n* (%)	85 (18.1)	59 (24.2)	26 (11.5)	0.0004
Hypertension, *n* (%)	365 (77.8)	186 (76.2)	179 (79.6)	0.386
Dyslipidaemia, *n* (%)	328 (69.9)	157 (64.3)	171 (76)	0.006
Carotid ATS, *n* (%)	239 (50.9)	127 (52)	112 (49.8)	0.623
Type 2 DM, *n* (%)	259 (55.2)	129 (52.8)	130 (57.8)	0.285
HF, *n* (%)	59 (12.6)	30 (12.3)	29 (12.9)	0.846
CAD, *n* (%)	106 (22.6)	53 (21.7)	53 (23.6)	0.635
AF, *n* (%)	331 (70.6)	172 (70.5)	159 (70.7)	0.966
TIA/minor stroke, *n* (%)	95 (20.3)	53 (21.7)	42 (18.7)	0.410
CKD (eGFR < 60 mL/min/1.73 m^2^), *n* (%)	207 (44.1)	113 (46.3)	94 (41.8)	0.323
COPD/asthma, *n* (%)	169 (36)	84 (34.4)	85 (37.8)	0.450
NAFLD, *n* (%)	85 (18.1)	44 (18)	41 (18.2)	0.957
ACEi/ARB, *n* (%)	312 (66.5)	155 (63.9)	156 (69.3)	0.215
ARNI, *n* (%)	34 (7.2)	19 (7.8)	15 (6.7)	0.640
SGLT2-i, *n* (%)	157 (33.4)	76 (31.1)	81 (36.0)	0.265
GLP1-RA, *n* (%)	107 (22.8)	55 (22.5)	52 (23.1)	0.883
Metformin, *n* (%)	199 (42.4)	98 (40.2)	101 (44.9)	0.300
DOAC, *n* (%)	313 (66.7)	165 (67.6)	148 (65.8)	0.671
VKA, *n* (%)	18 (3.8)	7 (2.9)	11 (4.9)	0.255
Β-blocker, *n* (%)	228 (72.0)	178 (72.9)	160 (71.1)	0.657
Statin, *n* (%)	222 (47.3)	104 (42.6)	118 (52.4)	0.033
PCSK9-i, *n* (%)	88 (18.8)	42 (17.2)	46 (20.4)	0.370
Anti-PLT, *n* (%)	136 (28.9)	71 (29.0)	65 (28.9)	0.960
Diuretic, *n (%)*	235 (50.1)	121(49.6)	114 (50.7)	0.815
MRA, *n* (%)	107 (22.8)	59 (24.0)	51 (22.8)	0.556
LAMA, *n* (%)	35 (7.5)	15 (6.1)	20 (8.9)	0.259
LAMA/LABA, *n* (%)	77 (16.4)	41 (16.8)	36 (16.0)	0.814
LAMA/LABA/ICS, *n* (%)	56 (11.9)	28 (11.5)	28 (12.4)	0.746

* performed by chi square test. Abbreviations: ATS, atherosclerosis; DM, diabetes mellitus; HF, heart failure; CAD, coronary artery disease; AF, atrial fibrillation; TIA, transient ischemic attack; CKD, chronic kidney disease; COPD, chronic obstructive pulmonary disease; NAFLD, non-alcoholic fatty liver disease; ACEi, angiotensin-converting enzyme inhibitor; ARB, angiotensin receptor blocker; ARNI, angiotensin receptor neprilysin inhibitors; SGLT2i, sodium/glucose cotransporter 2 inhibitor; GLP1-RA, GLP-1 receptor agonist; DOAC, direct oral anticoagulant; VKA, vitamin K antagonist; PCSK9-i, PCSK9 inhibitor; MRA, mineralocorticoid receptor antagonist; LAMA, long-acting muscarinic antagonist; LABA, long-acting beta agonist; ICS, inhaled corticosteroid.

**Table 2:** Comparison of baseline characteristics between treated and untreated group.

	Whole Population (*n* = 469)	Untreated Group (*n* = 244)	Treated Group (*n* = 225)	*p*
Age, years	74.8 ± 5	74.6 ± 4.3	75.0 ± 5.6	0.284
Gender (males), *n* (%)	330 (70.4)	174 (71.3)	156 (69.3)	0.639
CHA_2_DS_2_Vasc, score	3.8 ± 1.4	3.8 ± 1.3	3.9 ± 1.4	0.309
HAS-BLEED, score	2.8 ± 1.0	2.9 ± 0.9	2.6 ± 1.1	0.001
BMI, kg/m^2^	32.4 ± 6.2	32.2 ± 6.0	32.5 ± 6.3	0.673
ESS, score	10.6 ± 4.4	10.9 ± 4.7	10.4 ± 4.7	0.274
AHI, e/h	36.9 ± 16.1	36.7 ± 15.5	37.5 ± 17.7	0.576
TC 90, %	11.2 (3.0–32.7)	9.6 (2.9–32.6)	12.6 (3.4–34.4)	0.537
ODI, e/h	33.6 ± 15.9	32.3 ± 13.2	34.5 ± 18.3	0.084
Mean SpO_2_, %	91.8 ±3.2	92.0 ± 2.9	91.5 ± 3.4	0.065
Night-time HR, bpm	68.0 ± 12.3	69.0 ± 11.9	66.9 ± 12.5	0.056
SBP, mmHg	137.0 ± 13.5	137.4 ± 13.0	136.7 ± 14.0	0.572
DBP, mmHg	78.3 ± 10.0	78.3 ± 9.9	78.4 ± 10.0	0.917
Day-time HR, bpm	69.8 ± 10.9	69.6 ± 10.3	69.9 ± 11.6	0.792
HOMAi, score	4.9 ± 3.1	5.1 ± 3.1	4.6 ± 3.1	0.080
HbA1c, %	6.9 ± 1.3	6.8 ± 1.0	6.9 ± 1.5	0.209
LDL-C, mg/dL	117.9 ± 33.9	119.8 ± 34.2	115.8 ± 33.6	0.208
TGRs, mg/dL	126.1 ± 53.2	129.6 ± 57.9	122.4 ± 47.3	0.144
Creatinine, mg/dL	1.1 ± 0.3	1.2 ± 0.3	1.1 ± 0.3	0.008
eGFR, ml/min/1.73 m^2^	62.7 ± 17.5	61.2 ± 17.9	64.4 ± 16.9	0.048
Uric acid, mg/dL	6.7 ± 1.4	6.8 ± 1.3	6.7 ± 1.5	0.264
HCT, %	42.9 ± 5.3	43.1 ± 5.4	42.6 ± 5.3	0.312
Hb, g/dL	13.7 ± 1.7	13.7 ± 1.7	13.7 ± 1.7	0.977
PLTs, u/µL	248.1 ± 80.2	252.0 ± 81.8	243.4 ± 78.3	0.267
hs-CRP, mg/L	3.9 ± 3.4	3.7 ± 3.6	3.7 ± 5.2	0.874
Mallampati, score	2.6 ± 1.0	2.4 ± 1.0	2.9 ± 0.9	0.0001

**Table 3:** Comparison of vascular function parameters at baseline between treated and untreated group.

	Whole Population (*n* = 469)	Untreated Group (*n* = 244)	Treated Group (*n* = 225)	*p*
IMT dx, mm	1.02 ± 0.2	1.03 ± 0.2	1.02 ± 0.2	0.423
IMT sn, mm	1.02 ± 0.2	1.02 ± 0.2	1.02 ± 0.1	0.671
ABI dx	1.02 ± 0.2	1.03 ± 0.2	1.0 ± 0.2	0.374
ABI sn	1.04 ± 0.2	1.04 ± 0.2	1.04 ± 0.2	0.857
AI, %	40.2 ± 9.2	40.4 ± 9.6	39.9 ± 8.9	0.531
PP, mmHg	42 ± 13.3	42.1 ± 13.4	42.1 ± 13.5	0.955
PWV, m/s	9.8 ±1.7	9.7 ± 1.7	9.8 ± 1.6	0.556
AP, mmHg	29.8 ± 9.3	30.2 ± 9.7	29.4 ± 8.9	0.342
c-SBP, mmHg	109.0 ± 16.6	109.5 ± 15.5	109.1 ± 15.7	0.953
c-DBP, mmHg	62.6 ± 9.9	62.5 ± 10.5	62.6 ± 9.4	0.917
RHi, score	1.5 ± 0.4	1.6 ±0.4	1.5 ± 0.4	0.218

**Table 4:** Vascular function parameters at baseline and follow-up of the study population.

	Baseline	Follow-Up	*p*
AI, %	40.2 ± 9.2	36.2 ± 8.1	0.0001
PP, mmHg	42.1 ± 13.3	38.3 ± 12.3	0.0001
PWV, m/s	9.8 ±1.7	8.2 ± 1.5	0.0001
AP, mmHg	29.8 ± 9.3	27.4 ± 8.7	0.0001
c-SBP, mmHg	109.1 ± 16.6	103.8 ± 15.2	0.0001
c-DBP, mmHg	62.6 ± 9.9	60.4 ± 9.7	0.0001
RHi	1.5 ± 0.4	2.1 ± 0.4	0.0001

Abbreviations: PWV, pulse wave velocity; AI, augmentation index; PP, pulse pressure; AP, augmentation pressure; c-SBP, central systolic blood pressure; c-DBP, diastolic blood pressure; RHi, reactive hyperaemia index.

**Table 5:** Changes in major parameters between baseline and follow-up according to CPAP use.

	Untreated Group (*n* = 244)	Treated Group (*n* = 225)	*p* *
Λ BMI, kg/m^2^	−0.7 (−1.2/−0.3)	−0.7 (−1.6/−0.4)	0.081
Λ ESS, score	−6.3 (−9.0/−2.0)	−7.2 (−9.0/−4.0)	0.014
Λ AHI, e/h	−3.8 (−3.8/6.8)	−26.3 (−34.7/−16.4)	0.0001
Λ TC 90, %	−4.9 (−13.3/−1.2)	−10.2 (−25/−2.0)	0.0001
Λ ODI, e/h	−3.40 (−4.2/6.4)	−23.7 (−37.4/−13.5)	0.0001
Λ mean SpO_2_, %	3 (1.0/3.0)	3 (1.0/5.0)	0.001
Λ HOMAi, score	−1.1 (−2.4/−0.8)	−1.4 (−2.2/−0.6)	0.009
Λ eGFR, mL/min/1.73 m^2^	−6.0 (−8.2/−2.8)	6.4 (3/11.7)	0.0001
Λ uric acid, mg/dL	−0.4 (−1.3/−0.1)	−0.5 (−1.3/−0.2)	0.126
Λ HCT, %	−0.3 (1.7/1.2)	−0.3 (−2.0/1.2)	0.818
Λ hs-CRP, mg/L	−0.4 (−1.2/0.2)	−1.4 (−2.2/−0.5)	0.0001
Λ PWV, m/s	−1.0 (−1.7/−0.8)	−1.6 (−2.1/−1.3)	0.0001
Λ RHi, score	0.4 (0.3/0.5)	0.6 (0.5/0.7)	0.0001

* Performed by Mann–Whitney test. Abbreviations: BMI, body mass index; ESS, Epworth sleepiness scale; AHI, number of episodes of apnoea and hypopnoea/h; TC90, % of time with spO_2_ < 90%; ODI, number of episodes of desaturation > 3%/h; HOMAi, homeostatic model assessment index; e-GFR, estimated glomerular filtration rate (CKD-EPI); HCT, haematocrit; hs-CRP, high-sensitivity CRP; PWV, pulse wave velocity; RHi, reactive hyperaemia index.

**Table 6:** Simple linear regression analysis between Δpwv and Δrhi and δ of other covariates in the treated group.

	Δ PWV (r/p)	Δ RHi (r/p)
Δ BMI, g/m^2^	0.032/0.479	−0.021/0.664
Δ ESS, *score*	0.029/0.524	−0.097/0.047
Δ AHI, e/h	0.344/0.0001	−0.251/0.0001
Δ TC 90, %	0.048/0.379	−0.103/0.007
Δ ODI, e/h	0.253/0.0001	−0.248/0.0001
Δ mean SpO_2_, %	−0.043/0.491	0.023/0.727
Δ HOMAi, score	0.010/0.857	−0.021/0.727
ΔeGFR, ml/min/1.73 m^2^	−0.095/0.005	0.037/0.480
Δ uric acid, mg/dL	0.193/0.0001	−0.309/0.0001
Δ HCT, %	0.070/0.124	−0.098/0.041
Δ hs-CRP, mg/L	0.211/0.0001	−0.143/0.012

Abbreviations: BMI, body mass index; ESS, Epworth sleepiness scale; AHI, number of episodes of apnoea and hypopnoea/h; TC90, % of time with spO_2_ < 90%; ODI, number of episodes of desaturation > 3%/h; HOMA index, homeostatic model assessment index; e-GFR, estimated glomerular filtration rate; HCT, haematocrit; hs-CRP, high-sensitivity CRP; PWV, pulse wave velocity; RHi, reactive hyperaemia index.

**Table 7:** Stepwise multivariate linear regression analysis between Δpwv and Δrhi as dependent variable, and δ of different covariates.

Δ of PWV as Dependent Variable				Δ of RHi as Dependent Variable			
	R^2^ Partial (%)	R^2^ Total (%)	*p*		R^2^ Partial (%)	R^2^ Total (%)	*p*
Δ AHI, e/h	35.8	35.8	0.0001	Δ uric acid, mg/dL	27.0	27.0	0.0001
Δ ODI, e/h	10.4	46.2	0.0001	Δ AHI, e/h	13.0	40.0	0.0001
Δ hs-PCR, mg/L	5.5	51.7	0.0001	Δ ODI, e/h	7.0	47.0	0.0001
Δ uric acid, mg/dL	2.5	54.2	0.0001	Δ hs-PCR, mg/L	2.0	49.0	0.004
Δ eGFR, mL/min/1.73 m^2^	1.2	55.4	0.009	Δ ESS, score	1.0	50.0	0.028
----	----	----	----	Δ HCT, %	0.7	50.7	0.043
----	----	----	----	Δ TC 90, %	0.2	50.9	0.048

Abbreviations: AHI, number of episodes of apnoea and hypopnoea/h; ODI, number of episodes of desaturation > 3%/h; hs-CRP, high-sensitivity CRP; eGFR, estimated glomerular filtration rate; ESS, Epworth sleepiness scale; HCT, haematocrit; TC90, % of time with spO_2_ < 90%.

## Data Availability

The raw data supporting the conclusions of this article will be made available by the authors, without undue reservation.
